# Performance evaluation of friction stir welding using machine learning approaches

**DOI:** 10.1016/j.mex.2018.09.002

**Published:** 2018-09-06

**Authors:** Shubham Verma, Meenu Gupta, Joy Prakash Misra

**Affiliations:** National Institute of Technology Kurukshetra, India

**Keywords:** Modelling of Friction Stir Welding Process, Ultimate tensile strength, Gaussian process regression, Support vector machining, Multi-linear regression, Pearson VII, Radial based kernel function

## Abstract

The aim of the present study is to evaluate the potential of sophisticated machine learning methodologies, i.e. Gaussian process (GPR) regression, support vector machining (SVM), and multi-linear regression (MLR) for ultimate tensile strength (UTS) of friction stir welded joint. Three regression models are developed on the above methodologies. These models are projected to study the incongruity between the experimental and predicted outcomes and preferred the preeminent model according to their evaluation parameter performances. Out of 25 readings, 19 readings are selected for training models whereas remaining is used for testing models. Input process parameters consist of rotational speed (rpm), and feed rate (mm/min) whereas UTS is considered as output. Two kernel functions i.e. Pearson VII (PUK) and radial based kernel function (RBF) are used with both GPR and SVM regression. It is concluded that the GPR approach works better than SVM and MLR techniques. Therefore, GPR approach is used successfully for predicting the UTS of FS welded joint.

• The aim of the present study is to evaluating the friction stir welding process using sophisticated machine learning methodology, i.e. Gaussian process (GP) regression, support vector machining (SVM) and multi-linear regression (MLR).
• Three models are projected to study the incongruity between the experimental and predicted outcomes and preferred the preeminent model according to their evaluation parameter performances. Two kernel functions i.e. Pearson VII (PUK) and radial based kernel function (RBF) are used with both GPR and SVM regression.
• GPR approach works better than SVM and MLR techniques. Therefore, GPR approach is used successfully for predicting the UTS of FS welded joint.

## Graphical abstract

**Figure:**
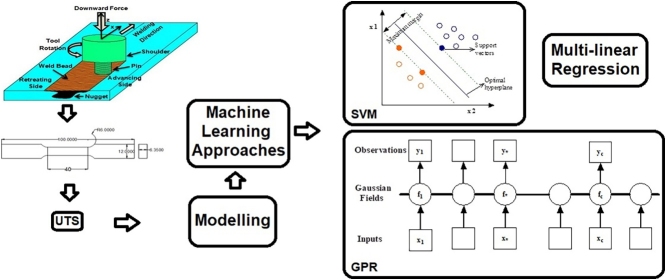

**Specification table**

**Table:** 

Subject Area:	*Engineering*
More specific subject area:	*Advanced Manufacturing Technique*
Method name:	*Modelling of Friction Stir Welding Process*
Name and reference of original method :	*Friction Stir Welding* *Thomas, W. M.; Nicholas, E. D.; Needham, J. C.; Murch, M. G.; Templesmith, P. & Dawes, C. J. (1991). Patent 9125978.8* *Mishra, R. S. & Mahoney, M. W. Friction stir welding and processing, ASM International, (2008).* *Verma, S., Gupta, M., & Misra, J.P., Friction Stir Welding of Aerospace Materials: A state of Art Review. DAAAM International Scientific Book, (2016)135-150.* *Verma, S., & Misra, J., A critical review of friction stir welding process. DAAAM International Scientific Book, (2015) 249-266.* *Machine Learning Approach* *Na, M. G., Kim, J. W., Lim, D. H., & Kang, Y. J., Residual stress prediction of dissimilar metals welding at NPPs using support vector regression. Nuclear Engineering and Design, 238(7), (2008)1503-1510.* *Wang, Y., Sun, Y., Lv, P., & Wang, H., Detection of line weld defects based on multiple thresholds and support vector machine. NDT & E International, 41(7), (2008) 517-524.* *C. E. Rasmussen & C. K. I. Williams, Gaussian Processes for Machine Learning, the MIT Press, 2006.*
Resource availability:	*Vertical Milling Machine* *WEKA 3.8 (Data Mining Software)*

## Method details

**Fig. 1:**
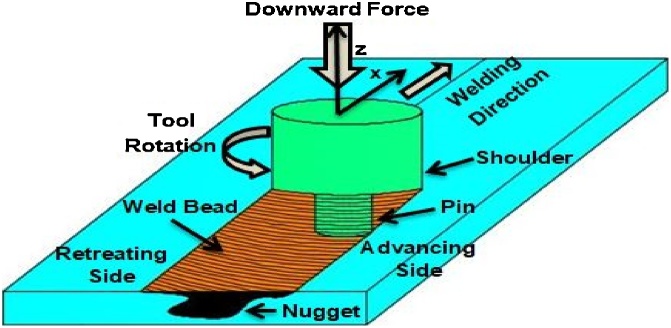
Process principle of FSW.

The demand of aluminium alloys is increasing in the area of aerospace, shipbuilding, automotive, transport, military and other many industries owing to their unique features, i.e., high strength to weight ratio, high formability, excellent corrosion resistance, etc. However, as similar to ferrous alloys the joining of aluminium alloys by conventional processes are very problematic due to high thermal conductivity, aluminium oxide formation, high thermal expansion, hydrogen solubility, etc. [1]. Aluminium has high thermal and electrical conductivity so more intense heat to be employed during fusion or resistance welding of this metal. This results in variation of mechanical and metallurgical properties of the joint changed. Furthermore, aluminium has high coefficient of thermal expansion and therefore tacking is mandatory before welding operation to make the weld uniform [2]. Sometimes due to the occurrence of solidification cracking during fusion welding of aluminium alloys, these alloys are considered as non-weldable alloys [3]. To improve the weld characteristics, Friction stir welding- a revolutionary welding technique was introduced by The welding Institute (TWI) in the year 1991. FSW process omitted arc-welding problems of aluminium alloys without change of phase of base metal or without reaching melting point. This revolutionary technique has a capability of joining thin and thick materials with a less skilled operator. FSW is a solid-state welding technique that produces joint with superior mechanical and metallurgical properties as compared to fusion welding process [4]. On the basis of its benefits, this technique still has not been fully commercialized around the world owing to high equipment cost, lack of industries standards and specification and design allow ability. The process principle of FSW is depicted in Fig. 1. Weld is produces with a non-consumable rotating tool with specially design pin and shoulder. The friction between the tool and workpiece generates heat that softens the material around the pin and owing to rotational and translational movement of tool the joint is produced. It can also be treated as green technology as it is free of any filler material or shielding gases [5]. In FSW, there is no need to melt the metal by arc for joining. FSW process is controlled by various process parameters like rotational speed, transverse speed, tilt angle, dwell time etc. These parameters affect the quality of the weld. So it is important to gather the detailed information of these welding process parameters which influence the quality of the weld.

Numerous publication has been published on this technique over the past 25 years. Patel et al. [6] studied the superplastic behavior of FS processed aluminium alloys. It is found that, superplasticity significantly depends upon the tool related parameters, machine-related parameters, heat distribution, cooling rate and strain rate. In addition, application of superplastic behavior in aviation and automotive industries are discussed. Patel et al. [7] reveal the effect of polygon pin profile on the friction stir processing of AA7075. The non-uniform microstructure was observed in the stir zone for all polygon pin profiles. The maximum tensile strength was obtained by using a square pin profile. Patel et al. [8] explained the effect of tool pin profiles on the temperature distribution during friction stir processing of AA7075. It was observed that the pentagon tool pin profile generates more heat during plunging. Moreover, tool shoulder has more influence on the heat generation during the process as compared to pin. Most of the researchers have focussed on mechanical and metallurgical properties of the FSW joint and few researchers are tried to evaluate the performance of FSW on different materials using different modeling and optimization technique. Okuyucu et al. [9] investigated the mechanical properties of FS welded joint by using artificial neural network modeling (ANN). Rajakumar and Balasubramanian [10] employed response surface methodology (RSM) for optimizing the process parameters of FSW. Shojaeefard et al. [11] employed ANN modeling for finding the correlation between the input and output process parameters. Tansel et al. [12] used genetically optimized neural network systems (GONNS) for modeling the FSW process. Ghetiya et al. [13] used Taguchi’s Tbased grey rational approach for optimizing the input parameters for FS welded AA8011. It is observed that maximum tensile strength is obtained at a tool diameter of 14 mm, the transverse speed of 80 mm/min, and a spindle speed of 1400 rpm respectively. Na et al. [14] employed tungsten arc welding for joining dissimilar welding metal plates. They used SVR model for predicting the residuals stresses of joint. It is concluded that these models are very much precise in predicting the experiment outcomes. Another researcher Wang et al. [15] observed that SVM more precisely classify the defect and non-defective features of the weld. Pal and Deswal [16] used gaussian process regression (GPR) approach for predicting water-engineering problems. Apart from SVM and GPR, researchers mostly used ANN models for predicting the performance of manufacturing processes [[17], [18], [19]]. Subsequently, hardly a work has been published on modeling of the tensile strength of FS welded AA6082 using machine learning approaches i.e. GPR and SVM. The present study investigates the ultimate tensile strength of FS welded AA6082 and examines the potential of machine learning technique i.e. GPR, SVM, and MLR. In addition, GPR model results are compared with SVM and MLR model results.

## Experimental procedure

**Fig. 2:**
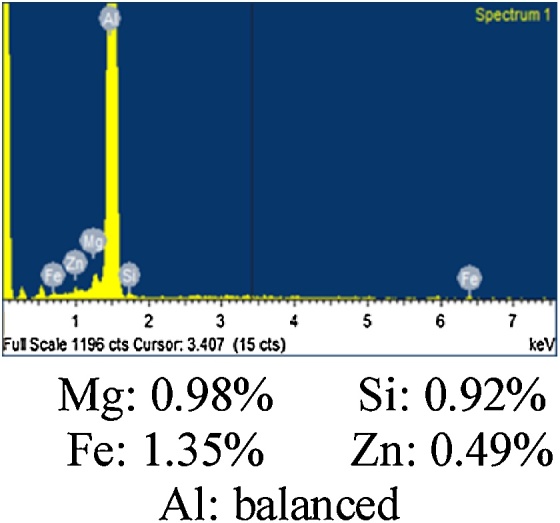
EDX analysis of 6082 aluminium alloy.

**Fig. 3:**
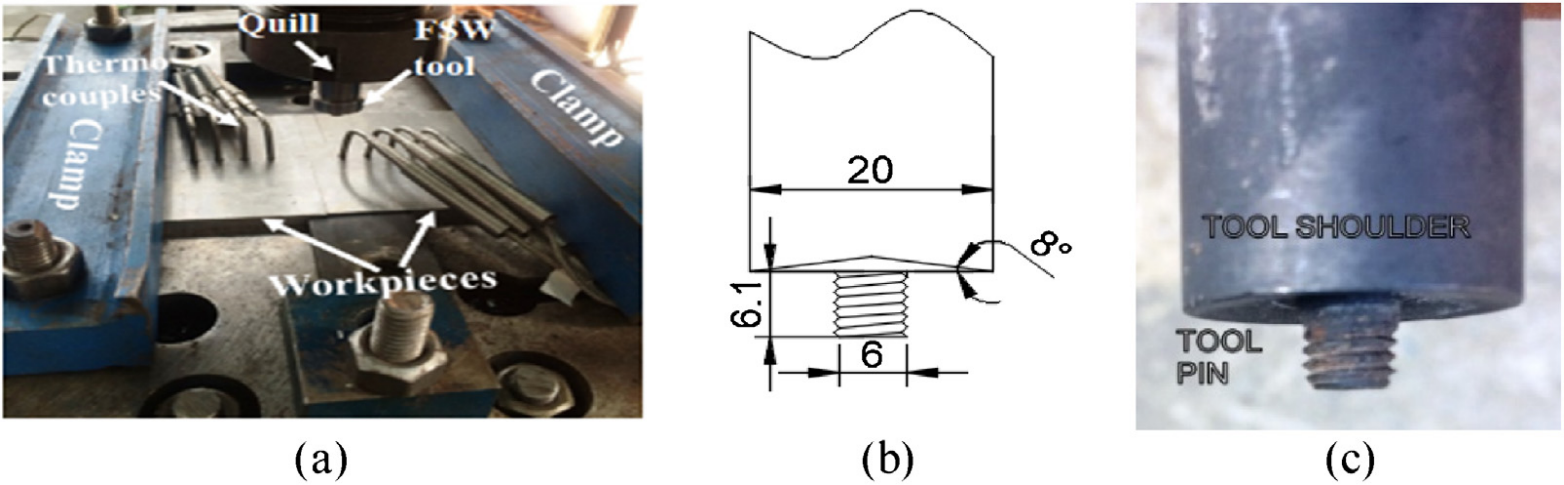
a) Fabricated fixture b) Schematic diagram for a tool (all dimensions are in mm) c) Fabricated tool.

**Fig. 4:**
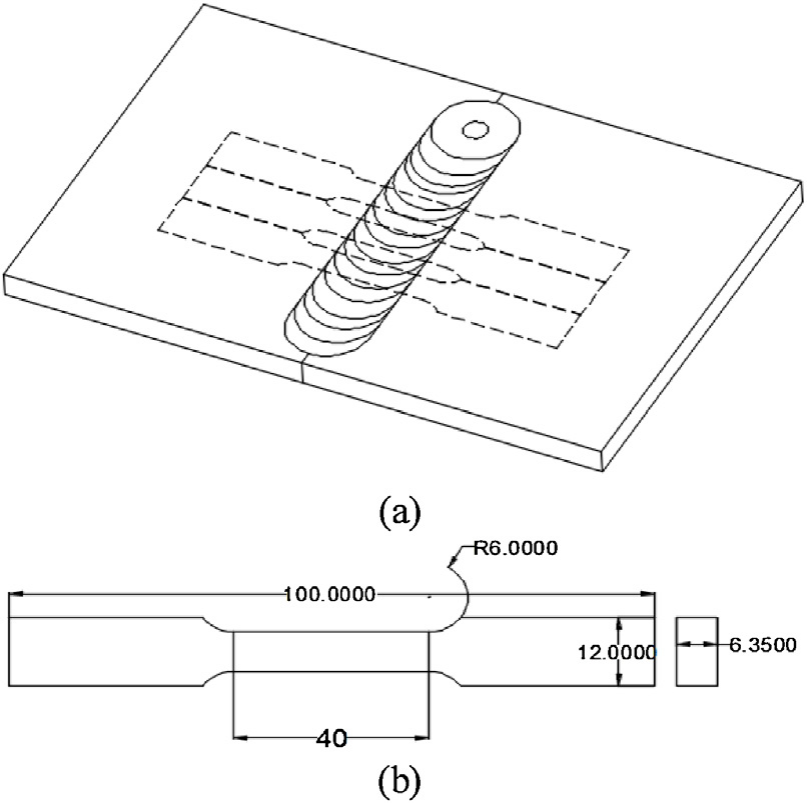
a) Schematic diagrams how to cut tensile specimen b) Schematic diagram of tensile specimen.

The material in this study is AA6082 which in the form of a rolled sheet of 6.35 mm thickness. The chemical composition of the given material is carried out by EDX analysis as shown in Fig. 2. In the present study, the experiments are designed on the basis of full factorial design. In which five levels of rotational and transverse speeds are used for fabricating the joint at a 2° tilt angle and 30 s dwell time. Table 1 reveals the input and output process parameters for the present study. The total number of experiments for five levels and two factors are 25 i.e. 5^2^. Every experiment is conducted with two replications for eliminating human error during the process. A vertical milling machine is used for conducting the experiments by fabricating tool and fixture as depicted in Fig. 3. The tool is made up of H13 die steel with a hardness of 54-56HRC. A tool having 20 mm diameter, 6.1 mm pin length with 6 mm pin diameter. Two plates of dimensions 100 mm × 80 mm × 6.35 mm are butt-welded at the right angle to the rolling direction. Fig. 4(a) shows the schematic diagram for how to cut tensile test specimen and schematic diagram of the tensile specimen. Firstly, the rectangular strips (150 × 12 × 6.35 mm) are cut from the welded sample on a power hacksaw. Afterward, these strips are converted into tensile specimen on the milling machine with the help of end milling cutter according to ASTM E8M-04 standard in a direction right angle to the welding direction. Next, these specimens are evaluated on a universal testing machine (UTM) for evaluating the ultimate tensile strength (UTS) of FS welded joint Fig. 4(b) illustrates the photographic view and dimension of the tensile specimen.

**Table 1:** Experimental design with outcomes.

S. No	Factor 1 Rotational Speed (rpm)	Factor 2 Feed Rate (mm/min)	UTS (MPa)
1	500	20	178
2	500	40	170
3	500	63	152
4	500	80	135
5	500	100	79
6	710	20	199
7	710	40	192
8	710	63	181
9	710	80	160
10	710	100	110
11	1000	20	220
12	1000	40	216
13	1000	63	203
14	1000	80	182
15	1000	100	143
16	1400	20	238
17	1400	40	242
18	1400	63	225
19	1400	80	193
20	1400	100	170
21	2000	20	215
22	2000	40	242
23	2000	63	260
24	2000	80	220
25	2000	100	192

## Modeling of tensile strength

In the present study, we used machine-learning techniques, namely, GPR, SVM and MLR for predicting the friction stir welding process. GPR, SVM, and MLR regression mathematical models are developed for the better of the ultimate tensile strength of the FS welded AA6082 joint. Then, the most suitable model is proposed on the basis of performance of model and graph analysis. Experimental outcomes are divided into two sets i.e. one set consist of training set data and other set consist of testing data set. Out of 25 observations, 83% data is randomly selected for the training set and remaining selected for testing set. With the use of training set data, different regression models are developed using machine learning approaches i.e. GPR, SVM and MLR. Two kernel functions i.e. Pearson VII (PUK) and radial based kernel function (RBF) are used with both GPR and SVM regression Afterward, adequacy of each developed model is checked on testing data set.

### Gaussian process regression

**Fig. 5:**
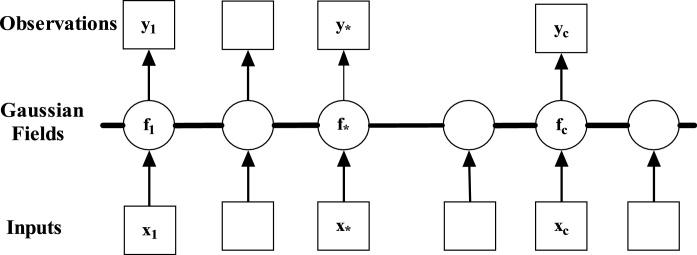
Graphical representation of GPR [21].

Gaussian process models are assuming a directional contingency between the input (x) and output (y) of the process. These models also pronounce the conditional distribution p (y/x). In GPR models, each observation provides the information of the adjacent observation value. It is the collection of random variables [20]. The graphical representation of GPR is shown in Fig. 5. The box shows the observed variables where the circles represent the unknown values. The line is the connection node between the various observed values. The observed values are tentatively independent upon the other nodes corresponding the values of f (latent variable). This is because of the degradation properties of the GPR. The main assumption of GPR is that the y is given by y = f(x) + ε, where ε ∼ N (0, σ ^2^). In the GPR, for each input values, there is a supplementary variable f (x). In the current study, ε is the observational error which is independently and identically distributed with variance σ_n_^2^. The observation becomes

(1) $$Y=\;\left({\text{y}}_{1}\ldots \;{\text{y}}_{\text{n}}\right)∼\;\text{N}\;\left(0,\;\text{K}\;+{\text{σ}}^{2}\text{I}\right)$$

Where, I is the identity matrix and K_ij_ = k (x_i,_ x*j*)

Because Y/X ∼ N (0, K + σ ^2^I) is normal and its training and test data of p(Y_*_/Y, X, X_*_). Then one has Y_*_/Y, X, X_*_ ∼ N (μ, Σ), where

(2) $$\mu \;=\;\text{K}\;\left(\text{X}*,\;\text{X}\right){\left(\text{K}\left(\text{X},\;\text{X}\right)+\;{\text{σ}}^{2}\text{I}\right)}^{-1}\text{Y}$$

(3) $$\text{Σ}=\text{K}\left(\text{X}*,\;\text{X}*\right)-\text{σ}{\;}^{2}\text{I}-\text{K}\;\left(\text{X}*,\;\text{X}\right)\;{\left(\text{K}\left(\text{X},\;\text{X}\right)\;+{\text{σ}}^{2}\text{I}\right)}^{-1}\text{Y}\;{\left(\text{K}\left(\text{X},\;\text{X}\right)\;+{\text{σ}}^{2}\;\text{I}\right)}^{-1}\;\text{K}\;\left(\text{X},\;\text{X}*\right)$$

Let us assume n and n_*_ are the training and testing data points then K (X, X_*_) represents the n x n matrix of the covariance estimated at each set of training and testing data points. In the same manner, this is also correct for K (X, X), K(X_*_, X_*_) and K(X_*_, X). The covariance matrix K (where K_ij_ = k (x_i,_ x*j*)) is generated with the help of covariance function. These covariance functions are similar as the use of kernel function in SVM. If the values of kernel k and noise σ ^2^ are known, then Eqs. (2) and (3) are sufficient for interpretation. The minimization of negative log-posterior [21]:

(4) $$\text{P}\;\left({\text{σ}}^{2}\text{k}\right)=\;½\;\left({\text{y}}^{\text{T}}\;{\left(\text{K}+\;{\text{σ}}^{2}\text{I}\right)}^{-1}\text{y}\right)+\;½\;\text{log}\;|\text{K}+\;{\text{σ}}^{2}\text{I}|\;-\;\text{log}\;\text{p}\;\left({\text{σ}}^{2}\right)\;–\text{log}\;\text{p}\;\left(\text{k}\right)$$

The hyper parameters are determined by differentiating Eq. (4) with respect to k and σ ^2^.

### Support vector machining

**Fig. 6:**
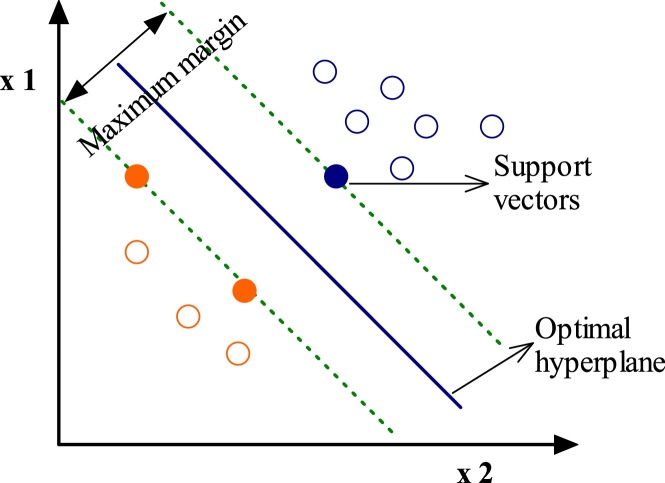
Hyperplanes of non-separable data set for SVM.

The SVM is a machine learning approach that is derived from statistical learning theory by Vapnik [22]. The goal of this approach is to determine the location of decision boundaries that produce the optimal separation of the classes. Support vector machine minimizes the generalization error. SVM are less time and cost taking technique for modeling the data with the least error as compared to ANN modeling [23]. The graphical representation of hyperplanes for the SVM approach is shown in Fig. 6. For more details regarding the SVM, readers are referred to Statistic learning theory by Vapnik in 1998.

Let n is the number of experiments and their training data set are represented $\left\{x_{j},y_{j}\right\}$, *j = 1, …, n,* where x ε R^N^ is represents the N-dimensional space and class label is y ε $\;\left\{-1+1\right\}$. If a vector **w** is exists (i.e. orientation determining vector for discriminating plane) than these training sets are called linearly separable variables and a scalar b used for determining the offset of discriminating plane from the origin as given below.

(5) $$y_{i}\;\left(W.x_{i}+b\right)-1\geq 0$$

For hypothesis space, the function is given by

(6) $$f_{w,b}=\text{sign}\;(\left(W.x+b\right)$$

### Multi-linear regression (MLR)

MLR is the conversion of data sets into logarithmic form. The relationship between the ultimate tensile strength of FS welded joint with rotational speed (R) and feed rate (F) is non-linear therefore, a following functional correlation may be supposed primarily.

(7) $$\text{UTS}\;\left(\text{MPa}\right)={\text{p}}_{\text{i}}{\text{R}}^{\text{a}}{\text{F}}^{\text{b}}$$

Whereas,

p_i_ *=* Proportionality constant.

Taking logs on both sides of Eq. (7) so to convert the equation into a linear form.

(8) $$\text{Log}\;\left(\text{UTS}\right)=\text{log}{\text{p}}_{\text{i}\;}+\text{a}\;\text{logR}+\log\text{F}$$

The above equation is multi-linear with two explanatory variables. For developing the multi-linear model, log (UTS) is taken as output parameters, two explanatory variables, namely log R, and log F are taken as input parameters. The output of MLR provides the values of p_i_, a and b and developed the equation in the form of Eq. (7).

(9) $$\text{UTS}=181.882\frac{{\text{R}}^{.0494}}{{\text{F}}^{.7888}}$$

## Details kernels of GPR and SVM

Machine learning approaches i.e. SVM and GPR consist of the use of various kernel functions. Numerous researchers suggested kernels on the basis of its performance i.e. radial basis kernels (RBF) and Pearson VII kernel (PUK) [[24], [25], [26]]. In this study, two kernel functions are used given below.

1 $\text{RBF}\;\left(\left(e{-}^{\gamma }\right|x_{i}-{\left(x_{j}\right|}^{2}\right)\;.$
2 Pearson VII function Kernal11+2xi−xj221ω−1/σ2ω.

Where γ, σ, ω are kernel specified parameters. Gamma (γ) is user-defined parameter for RBF kernel, which affect the classification accuracy of training data set. Sigma and omega are the parameters for PUK. The Pearson width is controlled by sigma (σ) parameter whereas omega (ω) is the trailing factor of the peak value when PUK is employed for curve fitting applications. GPR and SVM techniques are needs to be the establishment of suitable user-defined parameters selections upon which the precision of both modeling technique depends. Moreover, the selection of kernel and kernel-specified parameters, the SVM needs the creation of regularization parameters *C* and size of error-insensitive zone ε whereas GPR needs the determination of optimum values of the Gaussian noise level It is level after the target has been normalized/standardized/left unchanged. In the present study, a large number of hit and trails are carried out by using a different combination of user-defined parameters with GPR and SVM for the selection of user-defined parameters i.e. *C*, γ, σ, ω, and Gaussian noise. The criteria for choosing user-defined functions are based on the minimization of root mean square error and maximization of the correlation coefficient. On the basis of this criterion, optimum values of user-defined parameters are selected. Kernel-specific parameters for both regression techniques are same. Table 2 reveals the optimum values of user-defined parameters for PUK and RBF kernels based SVM and GPR. In this study, SVM_PUK, SVM_RBF, GPR_PUK, and GPR_RBF represents the Pearson VII kernel and radial basis kernel function based SVM and GPR. Root mean square error (RMSE) and correlation coefficient (CC) are used for checking the performance of both techniques.

**Table 2:** User-defined parameters for GPR and SVM using RBF and PUK kernel functions.

Kernels	GPR	SVM
PUK	Gaussian noise = 0.2, ω = 1, σ = 1	*C =* 0.2, ω = 1, σ = 1
RBF	Gaussian noise = 0.2, γ = 7	*C =* 0.2, γ = 7

## Results and discussion

**Fig. 7:**
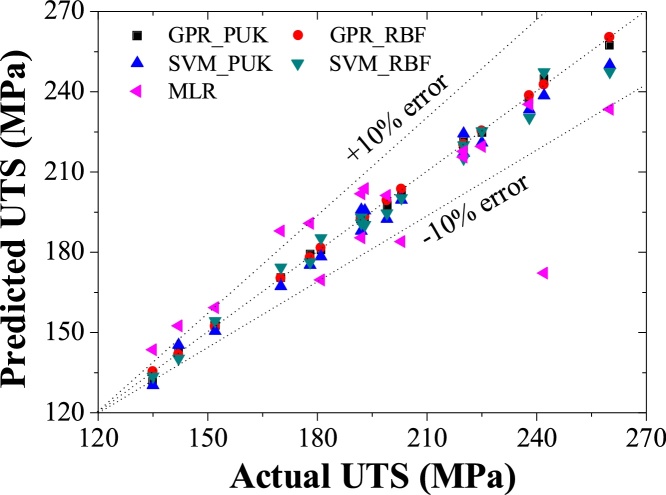
Actual vs predicted values of UTS using GP, SVM, and MLR using training data.

**Fig. 8:**
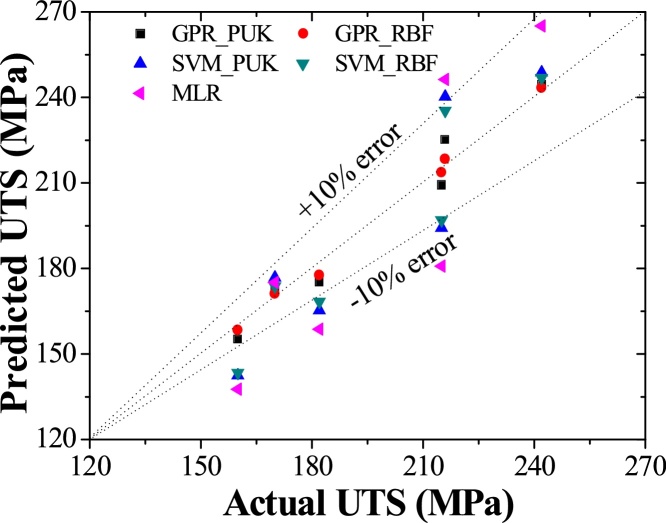
Actual vs predicted values of UTS using GP, SVM, and MLR using testing data.

Machine learning approaches i.e. GPR_PUK, GPR_RBF, SVM_PUK, SVM_RBF, and MLR are used to predict the values of UTS for each reading in training and testing data set as depicted in Table 3, Table 4. Two performance parameters i.e. coefficient of correlation (CC) and RMSE are practiced for evaluation of model outcomes for a better understanding of the obtained value of UTS through GPR_PUK, GPR_RBF, SVM_PUK, and SVM_RBF. Table 5 reveals the values of CC and RMSE for GPR, SVM and MLR approaches respectively. The graph for actual and predicted values of UTS for Pearson VII and RBF kernel based GP and SVM regression, as well as MLR using training and testing data sets, are depicted in Fig. 7, Fig. 8 respectively. Fig. 7 is for a data set of 19 training points and Fig. 8 is for the data set of 6 test points. Each graph consists of three lines i.e. one perfect line (line plotted at an angle of 45°) and others are error lines to examining the scattering of data around the perfect line. Each error lines are plotted in the limit of ± 10% error.

**Table 3:** Prediction results for UTS obtained by the entire model of training data.

S.No	RPM	Feed Rate	Actual UTS (MPa)	Predicted UTS (MPa) GPR_ PUK	Predicted UTS (MPa) GPR_ RBF	Predicted UTS (MPa) SVM_PUK	Predicted UTS (MPa) SVM_RBF	Predicted UTS (MPa) MLR
1	500	20	178	179.41	178.89	175.24	176.69	190.83
2	500	63	152	154.65	152.30	150.55	150.15	159.27
3	500	80	135	131.49	135.36	133.63	133.14	143.50
4	500	100	79	85.95	79.30	119.69	120.04	127.72
5	710	20	199	198.57	199.36	196.3	196.53	201.21
6	710	40	192	192.73	192.63	194.84	190.42	185.43
7	710	63	181	180.87	181.63	178.44	178.77	169.66
8	710	100	110	110.26	110.25	124.65	117.99	138.11
9	1000	20	220	220.42	220.36	222.65	217.51	215.55
10	1000	63	203	202.97	203.62	201.3	201.01	184.00
11	1000	100	142	142.90	142.50	142.81	140.27	152.4
12	1400	20	238	237.10	238.6	238.51	233.48	235.33
13	1400	63	225	224.76	225.36	221.01	222.81	219.55
14	1400	80	193	194.08	193.36	193.71	191.15	203.78
15	1400	100	170	170.65	170.30	169.56	168.15	188.00
16	2000	40	242	243.93	242.63	240.52	239.87	172.23
17	2000	63	260	257.32	260.35	250.01	244.92	233.45
18	2000	80	220	221.81	220.32	217.1	217.63	217.67
19	2000	100	192	192.87	192.85	187.94	190.52	201.89

**Table 4:** Prediction results for UTS obtained by the entire model of testing data.

S. No	RPM	Feed Rate	Actual UTS (MPa)	Predicted UTS (MPa) GPR_PUK	Predicted UTS (MPa) GPR_RBF	Predicted UTS (MPa) SVM_PUK	Predicted UTS (MPa) SVM_RBF	Predicted UTS (MPa) MLR
1	500	40	170	173.51	171.04	176.8	173.69	175.054
2	710	80	160	155.23	158.3	142.44	143.38	153.886
3	1000	40	216	225.3	214.3	245	241.32	232.222
4	1000	80	182	175.3	177.6	161.07	160.64	168.226
5	1400	40	242	244.5	243.27	248.8	246.81	265.004
6	2000	20	215	209.3	213.59	194.26	196.94	180.773

**Table 5:** Performance characteristics for UTS.

Machine learning approach	Training data set		Testing data set	
	CC	RMSE (MPa)	CC	RMSE (MPa)
GPR_PUK	0.9990	2.283	0.92	8.76
GPR_RBF	0.9995	1.692	0.97	5.94
SVM_PUK	0.9816	11.10	0.92	9.13
SVM_RBF	0.9828	10.63	0.95	6.19
MLR	0.9617	17.65	0.8621	22.40

**Fig. 9:**
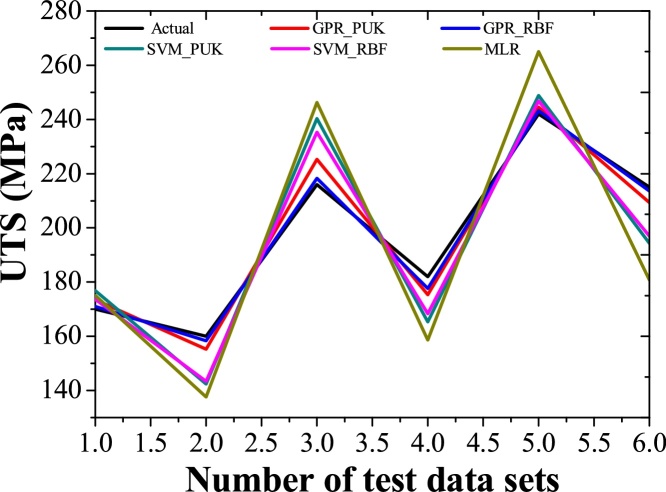
Variation in predicted values of UTS using the GPR, SVM, and MLR approaches in contrast to actual values of UTS.

**Fig. 10:**
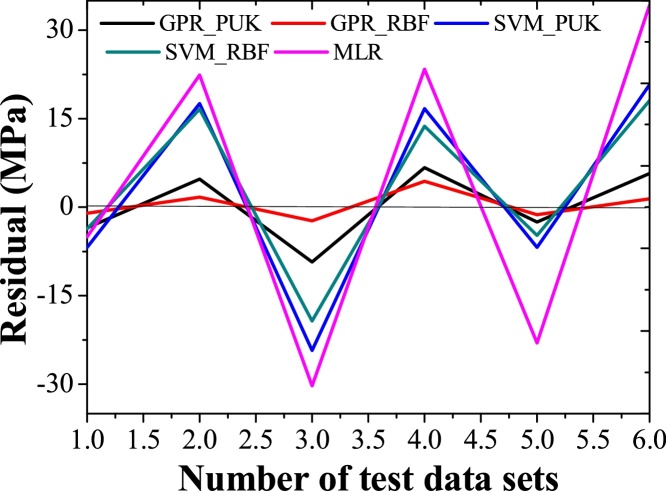
Residual from predicted and actual values of UTS using GPR, SVM and MLR approaches.

The evaluations of results suggest that GPR regression technique is in worthy agreement with the experimental outcomes than the SVM and MLR as indicated by Table 5. The predicted values of the training data set and test data set are agreed well with the experimental values. This shows that the GPR approaches are fruitful are in modeling the non-linear relationship between the UTS and input parameters. The coefficient of correlation .97 (RMSE 5.94 MPa) is attained by RBF kernel based GPR approach it shows slightly better performance in contrast to Pearson VII kernel based GPR technique Correlation coefficient value of 0.92 (RMSE 8.76 MPa). In MLR approach, the correlation coefficient i.e. 0.8621 with RMSE of 22.40 MPa is obtained. Keeping in the outlook of better performance of the GPR technique, a graph between test dataset numbers and UTS is drawn for all modeling techniques (Fig. 9). It is clear from the figure that predicted values provided by RBF and Pearson VII based GPR approach are in very close to actual values of UTS. It is observed that predicted values follow the same pattern as followed by the actual values. Fig. 10 shows the residual of predicted and actual values of UTS for the test data set. This shows that GPR has minor residual than the SVM and MLR.

## Conclusions

The current study explores the potential of GPR, SVM and MLR based regression model for predicting the UTS of FS welded AA6082 joint and compares their performances. From the evaluation of performance, it is concluded that RBF kernel based GPR regression technique works well (CC 0.97, RMSE 5.94 MPa) in comparison to SVM and MLR regression approaches for this data set. In addition, Pearson VII and RBF kernel based GPR works well than that of SVM and MLR regression for predicting the values of UTS of welding joint.
